# Glutathione S-transferase M1 and T1 genes deletion polymorphisms and blood pressure control among treated essential hypertensive patients in Burkina Faso

**DOI:** 10.1186/s13104-021-05658-w

**Published:** 2021-06-30

**Authors:** Herman Karim Sombié, Daméhan Tchelougou, Abdoul Karim Ouattara, Jonas Koudougou Kologo, Pegdwendé Abel Sorgho, Dogfunianalo Somda, Sakinata Yaméogo, Arsène Wendpagnangdé Zongo, Isabelle Touwendpoulimdé Kiendrebeogo, Enagnon Tiémoko Herman Donald Adoko, Albert Théophane Yonli, Florencia Wendkuuni Djigma, Patrice Zabsonré, Hassanata Millogo, Jacques Simporé

**Affiliations:** 1 Laboratory of Molecular Biology and Genetics (LABIOGENE), UFR/SVT, University Joseph Ki-Zerbo, 03 P.O. Box 7021, Ouagadougou 03, Burkina Faso; 2 Pietro Annigoni Biomolecular Research Center (CERBA), P.O. Box 364, Ouagadougou 01, Burkina Faso; 3 Saint Camille Hospital of Ouagadougou (HOSCO), 01 P.O. Box 444, Ouagadougou 01, Burkina Faso; 4 Faculty of Medicine, University Saint Thomas d’Aquin, P.O. Box 10212, Ouagadougou, Burkina Faso; 5 University Hospital Center-Yalgado Ouédraogo (CHUYO), 01 P.O. Box 676, Ouagadougou, Burkina Faso; 6 CERBA/LABIOGENE; University Joseph Ki-Zerbo, 01 BP 364, Ouagadougou 01, Burkina Faso

**Keywords:** Blood pressure control, Essential hypertension, GSTM1, GSTT1, Burkina Faso

## Abstract

**Objective:**

Glutathione S-transferases have been associated with experimental resistance to some drugs. The present study investigated the factors associated with blood pressure control in patients with essential hypertension, especially the role of *GSTT1* and *GSTM1* genes polymorphisms. This cross-sectional study in Burkina Faso consisted of 200 patients with essential hypertension and under treatment.

**Results:**

In the present study, 57.5% (115/200) of patients had their hypertension under control. No statistically significant difference was found between controlled and uncontrolled groups for anthropometric and biochemical parameters as well as for *GSTT1* or *GSTM1* gene polymorphisms (all *p* > 0.05). Current alcohol consumption (OR = 3.04; CI 1.88–6.13; *p* < 0.001), Physical inactivity (OR = 3.07; CI 1.71–5.49; *p* < 0.001), severe hypertension before any treatment (Grade III [OR = 3.79; CI 2.00–7.17; *p* < 0.001]) and heart damage (OR = 3, 14; CI 1.59–6.02; *p* < 0.001) were statistically more frequent in uncontrolled essential hypertensive patients than controlled hypertensive patients.

**Supplementary Information:**

The online version contains supplementary material available at 10.1186/s13104-021-05658-w.

## Introduction

Normalization of blood pressure (BP) in hypertensive patients significantly decreases the risk of stroke, heart diseases and improves patients' quality of life [1].

However, the achievement rates of the BP target values (Systolic Blood Pressure [SBP] < 140 mmHg and Diastolic Blood Pressure [DBP] < 90 mmHg) remain low in treated patients, estimated at 37.1% worldwide in 2010 and less than 10% in Sub-Saharan Africa in 2013 [2, 3]. Black people are the more susceptible subgroup to hypertension and its complications [4], and are more exposed to uncontrolled hypertension or use of multiple drugs to control their BP [5]. It therefore becomes crucial to better understand factors that affect BP control in order to minimize their effects. Many studies have looked for factors associated with hypertension control but the contribution of genetic factors is less studied. Glutathione S-transferase (GST) plays a crucial role in the detoxification mechanisms of drugs and xenobiotics [6]. Studies in both humans and animals have shown that polymorphisms which affect the expression of certain enzymes in the GST family also affect the effectiveness of certain drugs [7–10]. These results suggest that GST could affect the bioavailability of certain drugs which acts as GST enzyme substrate. To date, no study to our knowledge has evaluated the link between GST gene polymorphisms and response to antihypertensive drugs, although Glutathione S-transferases Mu1 deletion has been associated with resistant hypertension [11]. We previously investigated the link between Glutathione S-transferases Mu1 (*GSTM1*) and theta 1 (*GSTT1*) variants and the risk of developing essential hypertension and found that GSTT1-null genotype was associated with the risk of developing hypertension in Burkina Faso [12]. In the present study, we want to specifically determine the implication of GSTM1/GSTT1 deletion polymorphisms in BP control among the followed hypertensive patients in the same population. We hypothesis that the GSTM1/GSTT1 active variants with normal detoxification activity, could reduce the bioavailability of antihypertensive drugs and by doing so, GSTM1/GSTT1 deletion polymorphisms, in addition to their association with the risk of developing essential hypertension, could modulate the response to antihypertensive treatments, therefore the control of BP. Hence, we aim to determine the factors associated with BP control among hypertensive patients in Burkina Faso, especially identify the contribution of *GSTM1* / *GSTT1* deletion polymorphisms.

## Main text

### Methods

#### Study design

We conducted a cross-sectional study from July 15, 2017 to March 27, 2018, including 200 essential hypertensive patients followed in the cardiology department of Saint Camille Hospital of Ouagadougou (HOSCO), University Hospital Center Yalgado Ouédraogo (CHUYO) and the Medical Center of General Aboubacar Sangoulé Lamizana military Camp).

The study population consisted of subjects under antihypertensive treatments regardless of gender or social characteristics, aged from 18 to 70 years old.

Patients with secondary hypertension or no antihypertensive treatment, pregnant women and subjects not descendants from Burkina Faso were not included in this study.

Controlled blood pressure was defined as an average of SBP < 140 mmHg and DBP < 90 mmHg for all patients [13] during the last two consecutive medical visits under treatment.

### Samples and data collection

A standardized questionnaire was used to collect socio-demographic, lifestyle, clinical and biological data (see questionnaire in Additional file 3).

BP was measured using an electronic cuffed sphygmomanometer by cardiologist as described previously [14].

Body mass index (BMI) was used to classify patients as obese (≥ 30 kg/m^2^), overweight (25–30 kg/m^2^), normal weight (20–25 kg/m^2^) and underweight (≤ 20 kg/m^2^). We determined waist circumference (WC) and abdominal obesity in men was determined when WC ˃ 102 cm and in women when WC > 88 cm [15]. Family history of hypertension was determined in participants with at least one close family member being hypertensive before the age of 60 years.

Alcohol consumption corresponds to any consumption during the last 30 days preceding the survey as mentioned in the report of the STEPS survey in Burkina Faso in 2013 [16]. We distinguished 3 types of alcohol drinkers: heavy drinkers (more than 6 drinks on any day for men or more than 4 drinks on any day for women), intermediate drinkers (between 3 and 6 drinks on any day for men or between 2 and 4 drinks on any day for women) and moderate or occasional drinkers (2 drinks or less in a day for men and 1 drink or less in a day for women).

From each patient, venous blood sample was taken in EDTA tube and anticoagulant-free tube. Sera were directly used for biochemical analysis using *CYANExpert 130 analyzer*, and blood pellet were stored at -20 °C until DNA extraction.

### DNA extraction and genotyping

The salting out method as described by Miller and *al.* in 1988 was used to isolate genomic DNA from peripheral white blood cells [17].

Genotyping of the *GSTM1* and *GSTT1* genes has been previously described [14]. Briefly we performed multiplex PCR with the GeneAmp PCR system 9700 (Applied Biosystem, USA) in a reaction volume of 25µL including 10µL of Master Mix AmpliTaq Gold® (Applied Biosystems, USA), 7µL of nuclease-free water, 5µL of DNA and 1µL of each primer pairs for each gene (*β-globin, GSTM1, GSTT1*). After amplification, PCR products were migrated on ethidium bromide-stained 3% agarose gel during 45 mn, bands were visualized under UV light at 312 nm using the Geneflash revelation device (Additional file 1) and the generated data were interpreted as previously described [14].

### Statistical analysis

We used Statistical Package for Social Sciences (20.0) and Epi Info (6.0) for data analyses. To determine sample size, we have taken into account following values: 95% of two-sided confidence level, 80% of power, odds ratio more than 2.2, ratio of controlled BP to uncontrolled BP 1.1, the proportion of controlled BP patients group having *GSTM1-*null and *GSTT1-*null about 50%. We expressed quantitative variables and frequencies as mean ± standard deviation and percentage respectively and comparisons between groups were done with t-test and chi-squared test respectively. Difference was considered as statistically significant when *p* < 0.05.

## Results

### Characteristics of the study population

The Table 1 presents the general characteristics of the study population. The BP levels of participants under treatment allowed us to classify them into patients with controlled and uncontrolled hypertension. We showed that 115 (57.5%) had their BP under control.

**Table 1:** General characteristics of the study population according to hypertension control

Parameters	Total *n* = 200 (100%)	Controlled HTA *n* = 115 (57.5%)	Uncontrolled HTA *n* = 85 (42.5%)	*p* value
Gender (M/F)	71/129	40/75	31/54	0.88
Age (years)	54.06 ± 10.89	54.16 ± 11.1	53.95 ± 10.70	0.89
SBP (mmHg)	137.54 ± 16.84	126.74 ± 8.55	150.61 ± 15.06	< 0.001*
DBP (mmHg)	83.45 ± 14.40	78.77 ± 8.27	89.11 ± 17.87	< 0.001*
BMI (Kg/m^2^)	28.76 ± 6.38	29.14 ± 7.22	28.39 ± 5.32	0.40
WC (cm)	94.55 ± 13.17	94.17 ± 13.26	95.00 ± 13.13	0.66
Glucose (mM)	5.44 ± 0.96	5.58 ± 1.11	5.41 ± 1.01	0.51
HDL-c (mM)	1.56 ± 0.93	1.52 ± 0.47	1.61 ± 1.27	0.72
LDL-c (mM)	2.98 ± 1.00	2.82 ± 0.93	3.15 ± 1.05	0.19
Total Cholesterol (mM)	5.13 ± 0.99	4.88 ± 0.98	5.34 ± 0.97	0.1
Triglycerides (mM)	1.26 ± 0.94	1.13 ± 0.51	1.39 ± 1.23	0.28
Creatine (μM)	111.52 ± 94.42	101.47 ± 43.85	121.57 ± 126.48	0.40

Values are expressed as mean ± standard deviation for continuous variables; the statistical analyzes were made by the t test or the chi-square test; *: significant difference between the groups (p < 0.05); SBP, systolic blood pressure; DBP, diastolic blood pressure; WC, waist circumference; HDL-c, high density lipoprotein cholesterol; LDL-c, low density lipoprotein cholesterol; mM, millimolar; µM, micromolar

Regarding the socio-demographic and biochemical data, there was no significant difference between the controlled and the uncontrolled group (all *p* > 0.05).

Of the 200 patients under antihypertensive treatments, 99 patients were under monotherapy, 65 patients under bitherapy and 36 patients under Tritherapy (data not shown). Additional file 2.

### Influence of genetic variants of *GSTM1* and *GSTT1* on the control of blood pressure and essential hypertension

The Table 2 presents and compares the frequencies of *GSTM1* and *GSTT1* variants between the controlled and uncontrolled SBP groups, between the controlled and uncontrolled DBP groups and between the controlled and uncontrolled hypertension group. We did not find any significant difference between those groups (*p* > 0.05). Stratified Analysis by age and sex also showed no association (Additional file 3).

**Table 2:** Distribution of *GSTM1* and *GSTT1* genes variants according to control state of essential hypertension, SBP and DBP

Parameters	Genes-variants	Systolic Blood Pressure (SBP)			Diastolic Blood Pressure (DBP)			Hypertension		
		Controlled *n* = 126 (%)	Uncontrolled *n* = 74 (%)	*p* value	Controlled *n* = 152 (%)	Uncontrolled *n* = 48 (%)	*p* value	Controlled *n* = 115 (%)	Uncontrolled *n* = 85 (%)	*p* value
Monotherapy *n* = 110	*#GSTM1-active*	55 (74.32)	30 (83.33)		66 (76.74)	19 (82.14)		51 (76.12)	34 (79.07)	
	*GSTM1-null*	19 (25.68)	6 (16.77)	0.34	20 (23.26)	5 (17.86)	1.00	16 (23.88)	9 (20.93)	0.48
	*# GSTT1-active*	28 (37.84)	10 (27.78)		31 (36.05)	7 (35.71)		27 (40.30)	11 (25.58)	
	*GSTT1-null*	46 (62.16)	26 (72.22)	0.39	55 (63.95)	17 (64.29)	0.63	40 (59.70)	32 (74.42)	0.15
Bitherapy *n* = 55	*# GSTM1-active*	17 (60.71)	17 (70.83)		24 (61.54)	10 (64.71)		16 (57.14)	18 (66.67)	
	*GSTM1-null*	11 (39.29)	7 (29.17)	0.56	15 (38.46)	6 (35.29)	1.00	12 (42.86)	9 (33.33)	0.58
	*# GSTT1-active*	14 (50.00)	9 (37.50)		16 (41.03)	7 (41.18)		11 (39.29)	12 (44.44)	
	*GSTT1-null*	14 (50.00)	15 (62.50)	0.41	23 (58.97)	9 (58.82)	1.00	17 (60.71)	15 (55.56)	0.78
Tritherapy *n* = 35	*# GSTM1-active*	13 (61.91)	9 (64.28)		19 (70.37)	3 (45.45)		12 (60.00)	10 (66.67)	
	*GSTM1-null*	8 (38.09)	5 (35.72)	1.00	8 (29.63)	5 (54.55)	0.11	8 (40.00)	5 (33.33)	0.73
	*# GSTT1-active*	7 (33.33)	8 (57.14)		10 (37.04)	5 (54.55)		7 (35.00)	8 (53.33)	
	*GSTT1-null*	14 (66.67)	6 (42.86)	0.18	17 (62.96)	3 (45.45)	0.24	13 (65.00)	7 (46.67)	0.32
Total *n* = 200	*# GSTM1-active*	85 (67.46)	56 (75.68)		109 (71.71)	32 (69.64)		79 (68.70)	62 (72.94)	
	*GSTM1-null*	41 (32.54)	18 (24.42)	0.26	43 (28.29)	16 (30.36)	0.58	36 (31.30)	23 (27.06)	0.53
	*# GSTT1-active*	49 (38.89)	27 (36.49)		57 (37.50)	19 (41.07)		45 (39.13)	31 (36.47)	
	*GSTT1-null*	77 (61.11)	47 (63.51)	0.76	95 (62.50)	29 (58.93)	0.86	70 (60.87)	54 (63.53)	0.76
	*#GSTM1*(+)*/GSTT1*(+)	25 (19.84)	16 (21.62)		34 (24.34)	7 (14.58)		24 (20.87)	17 (20.00)	
	*GSTM1*(−)*/GSTT1*(+)	24 (19.05)	11 (14.87)	0.63	23 (15.13)	12 (25.00)	0.11	21 (18.26)	14 (16.47)	1.00
	*GSTM1*(+)*/GSTT1*(*−*)	60 (47.62)	40 (54.05)	1.00	75 (49.34)	25 (52.08)	0.37	55 (47.83)	45 (52.94)	0.71
	*GSTM1*(−)*/GSTT1*(−)	17 (13.49)	7 (9.46)	0.59	20 (13.16)	4 (8.33)	1.00	15 (13.04)	9 (10.59)	0.79

Values are expressed in numbers (percentages) and the comparison between groups was made using the chi-square test; *: significant difference between the groups (p < 0.05); #: reference; ( +): active; (−): null

### Research of non-genetic factors associated with the control of essential hypertension

The Table 3 presents and compares the frequencies of non-genetic factors that have been associated with hypertension control in previous studies. Our results showed that current alcohol consumption (OR = 3.04; CI 1.88–6.13; *p* < 0.001), physical inactivity (OR = 3.07; CI 1.71–5.49; *p* < 0.001), severe hypertension before any treatment (Grade III [OR = 3.79; CI 2.00–7.17; *p* < 0.001]) and heart damages, including left ventricular hypertrophy, left ventricular relaxation anomalies, left atrial hypertrophy and mitral insufficiency (OR = 3, 14; CI 1.59–6.02; *p* < 0.001) were more frequent in uncontrolled group than controlled group and differences were significant. Age and sex-stratified analysis showed that this association is specific to women and elderly subjects for current alcohol use, specific to men and elderly subjects for cardiac damages and specific to women for diabetes mellitus (Additional file 4).

**Table 3:** Bivariate analysis of non-genetic factors affecting blood pressure control in patients with essential hypertensive

Parameters	Controlled *n* = 115 (%)	Uncontrolled *n* = 85 (%)	OR	CI	*p* value
Sex					
Men/Women	40/75	31/54	0.92	0.51–1.16	0.88
Age					
≤ 45 years	22 (19%)	21 (25%)	1.38	0.70–2.73	0.38
46—55 years	44 (38%)	31 (36%)	0.92	0.51–1.65	0.88
56—65 years	28 (24%)	25 (30%)	1.29	0.68–2.43	0.42
≥ 66 years	21 (18%)	8 (9%)	0.46	0.19–1.10	0.10
Residence					
Rural/ Urban	31/84	15 /70	1.72	0.86–3.44	0.13
Behavioral factors					
Current alcohol use	34 (30%)	50 (59%)	3.04	1.88–6.13	< 0.001*
Current tobacco use	10 (8.6%)	7 (8%)	0.94	0.34–2.58	1
Low sodium diet	6 (5.2%)	7 (8%)	1.63	0.52–5.03	0.40
Lack of physical exercise	39 (34%)	52 (61%)	3.07	1.71–5.49	< 0.001*
Normal weight	35 (30%)	23 (27%)	0.84	0.45–1.57	0.63
Overweight and obesity	80 (70%)	62 (73%)	1.17	0.63–2.19	0.63
Central obesity	60 (52%)	48 (56%)	0.84	0.48–1.48	0.56
Grade hypertension					
Grade I	44 (39%)	14 (16%)	0.31	0.16–0.63	< 0.001*
Grade II	50 (43%)	32 (38%)	0.78	0.44–1.39	0.46
Grade III	21 (18%)	39 (46%)	3.79	2.00–7.17	< 0.001*
Personal history					
Heart involvement Yes/No	17/98	30/55	3.14	1.59–6.02	< 0.001*
Diabetes mellitus Yes/No	14/101	7/78	0.64	0.24–1.68	0.48
Asthma Yes/No	3/112	3/82	1.36	0.26–6.93	0.70
Taste Yes/No	6/109	6/79	1.37	0.42–4.43	0.76
Family history					
Hypertension Yes/No	70/45	56/29	1.24	0.69–2.22	0.55
Diabetes mellitus Yes/No	70/45	56/29	1.24	0.69–2.22	0.55
Treatment level					
Monotherapy	59 (51%)	40 (47%)	0.90	0.51–1.58	0.77
Bitherapy	35 (31%)	30 (35%)	1.21	0.67–2.18	0.54
Tritherapy	21 (18%)	15 (18%)	0.88	0.41–1.85	0.85
Professional status					
Household	45 (40%)	24 (32%)	0.61	0.33–1.11	0.13
Farmer	5 (4%)	2 (2%)	0.53	0.10–2.80	0.70
Official	24 (21%)	24 (32%)	1.49	0.77–2.86	0.24
Daily	1 (1)	1 (1%)	1.35	0.10–22.01	1
Unemployed	6 (5%)	5 (7%)	1.13	0.33–3.85	1
Retirement	9 (8%)	7 (9%)	1.05	0.37–2.96	1
Trader	20 (17%)	14 (21%)	1.09	0.51–2.3	0.84
Other	5 (4%)	8 (13%)	2.28	0.72–7.25	0.24

Values are expressed in numbers (percentages) and the comparison between groups was made using the chi-square test; *: significant difference between the groups (p < 0.05)

## Discussion

In this study, we investigate the factors associated with BP control in essential hypertensive patients from Burkina Faso. Our results showed that there was no significant difference between the controlled and the uncontrolled hypertension group by comparing the levels of biochemical parameters, suggesting that BP control is independent of blood glucose, cholesterol, triglycerides and creatinine levels in hypertensive patients.

There was also no significant difference between the control rates of patients under monotherapy and bitherapy or tritherapy, unlike some previous studies which have shown that monotherapy in Burkinabe [18] or multi-drug therapy in Brazilian [19] was associated with uncontrolled BP.

However, we found that alcohol consumption, physical inactivity, the high grade of initial hypertension before any medication and cardiac affections were associated with uncontrolled BP. The influence of alcohol consumption on antihypertensive therapy has long been studied. Stewart et al*.,* in a multi-ethnic cohort (including 76% non-Hispanic white, 12% Hispanic, 8% African American, and 4% other ethnic groups), showed that the reduction of alcohol consumption increase the antihypertensive drugs response and that the management of alcohol consumption must be considered as a major component of antihypertensive therapy in alcoholics [20]. Concerning physical exercise, many studies have consistently demonstrated its beneficial effects on hypertension. Diaz and Shimbo in a review, showed reductions in SBP and DBP up to 5–7 mmHg [21] and Pescatello in another review found that more frequent and long-term exercise leads to a more sustained reduction in BP, called exercise training response [22]. It is believed that the reduction in BP with physical activity is due to the attenuation of peripheral vascular resistance, which may be due to neurohormonal and structural responses [23]. Other mechanisms suggested in reducing BP through exercise include favorable changes in oxidative stress, inflammation, endothelial function, body mass, activity of the renin-angiotensin system, renal function, and insulin sensitivity [21].

Cardiac damages were found more in the uncontrolled group compared to the controlled group and this may be the cause or the effect of uncontrolled BP in essential hypertensive patients.

Our results also showed that there was no association between socio-demographic characteristics, residence areas and gender with BP control, unlike other studies which have shown that women had a higher control rate than men in African countries [24]. Our results are similar to those reported in Tanzania by Maginga et al*.,* who showed that age, gender, educational level, marital status, professional status and residency did not affect the control of hypertension [25]. However, unlike our study which found no association between obesity and the control of essential hypertension, that of Maginga et al*.,* showed that it was associated with a poor control rate in Tanzania.

Considering the genetic aspects, we investigated the influence of variants of the *GSTM1* and *GSTT1* genes on the control of essential hypertension. Genetic factors may influence the pharmacokinetics and pharmacodynamics (tissue or organ responsiveness) of drugs [26].

In our study, out of 99 patients, 96 used vasodilator drugs (Amlodipine, Nifedipine, Captopril, Ramipril, Enalapril) which reduced blood pressure by dilating or preventing constriction of the blood vessels [27]. The remaining 3 patients were using Atenolol, a beta blocker which binds to the beta receptors for adrenaline and norepinephrine, blocking their actions and thus promoting a slowing of the heart rate and drop in blood pressure [28]. In dual therapy, all patients used at least one vasodilator in addition to other classes of antihypertensive drugs such as beta blockers (14/65) and diuretics (39/65) which lower the blood pressure by increasing diuresis and thus reducing blood volume [29]. In triple therapy in 36 patients, 35 used two vasodilators in addition to a diuretic (32/35) or a beta blocker (2/35) or a central antihypertensive (1/35). Only one out of the 36 patients used a vasodilator in addition to a diuretic and a beta blocker. Altogether, vasodilators were the most used therapy (72.40%), followed by diuretics (21.36%), beta-blockers (5.4%) and centrally acting antihypertensive (0.30%). In the literature, to our knowledge, no direct link has been shown between these antihypertensive drugs used and the *GST* genes; however, associations have been demonstrated with the efficacy of other drugs in other pathologies and conditions. So in cancer cells, Gate et al*.,* demonstrated that *GST* often show high levels of expression when compared to normal cells [30] and this may contribute to increase detoxification of anticancer drugs [31]. It have been also shown that *GST* Through their detoxification activity, might play an important role in the protection against the toxic effect of the antimicrobial agents which leads bacteria to become resistant to antibiotics [32]. In Ghanaian HIV treated patients, homozygous deletion of *GSTM1* and *GSTT1* have been associated with CD4 + count rising above 350 cells/mm^3^ suggesting that patients with homozygous deletion have slower disease progression and better drug response [33]. Among *GST* genes, *GSTM1* and *GSTT1* are the most investigated in studies exploring genetic and drug response and they have been described as polymorphic in humans [34]. The most common polymorphisms of the loci of the *GSTM1* and *GSTT1* genes consist in the complete deletion of these genes (*GSTM1*-null and *GSTT1*-null) [35]. The GSTM1-null variant represents the complete or partial deletion of the GSTM1 gene and results in loss of function for GSTM1 enzyme. The GSTM1 locus has been mapped on chromosome 1p13.3 (GRCh38/hg38). Three different alleles have been identified in the same locus, including gene deletion, and two other mutations (GSTM1a and GSTM1b) that differ by C to G substitution at base position 534 [36]. Similarly, a deletion polymorphism in *GSTT1* leads to lack of enzyme activity. The human GSTT1 locus has been mapped on chromosome 22q11.23 (GRCh38/hg38). Regarding GSTT1, the null allele results from the homologous recombination of the left and right repeats of 403 bp, which results in a deletion of 54, 251 bps containing the entire GSTT1 gene. Whether it is GSTM1 or GSTT1, three distinct phenotypes can thus be observed in the population namely the “nonconjugators: 0/0”, the “low conjugators: 1/0” and “high conjugators: 1/1” [37]. The “nonconjugators” (homozygous deletion or null genotype) who lost GSTM1 or GSTT1 gene entirely, no longer have the capacity to conjugate the glutathione to the specific substrate, hence their accumulation in the organism. The “low conjugators” (heterozygotes) which have half-conserved the GSTM1 or GSTT1 gene have a reduced capacity for conjugation of the glutathione and “high conjugators” (without deletion) which have retained the gene in its entirety have a high conjugation activity and therefore elimination of substrates specific to the gene. The frequencies of GSTM null and GSTT1 null varies widely in different populations. Previous studies in Burkina Faso reported that approximately 28.75%–31.23% and 30%–55.76% of the population have the null variant of *GSTM1* and *GSTT1* respectively [12, 38], which fell into the range of allele frequency values registered in west African Nigeria (0.3 for GSTM1 null allele; 0.37 for GSTT1 null allele) [39] and other Black African populations [40]. *GSTM1* and *GSTT1* play a key role in the metabolism of certain drugs and xenobiotic through their participation in the second phase of xenobiotic metabolism. They facilitate the excretion of electrophilic compounds from cells by conjugating them to hydrophilic compounds with reduced glutathione [41, 42]. Thus the “conjugators” and “high conjugators” with their capacity of elimination could be subject to a decrease in the bioavailability of drugs which are substrates of *GSTM1* or *GSTT1*, therefore a decrease in the response to these drugs, hence the absence or decrease in BP control in those subjects. However our results showed that neither *GSTM1* nor *GSTT1* was associated with BP control. This at first sight indicates an absence of association between the active variants of these two genes and the low availability and efficacy of antihypertensive drugs. Some studies estimate that other members of the GST enzyme family must have compensated for the absence of a functional enzyme in the null genotype subjects [43, 44], which leads to the same level of activity of GST enzymes both in the GSTM1/GSTT1 null and active genotypes.

## Conclusion

In this study, we have not detected any apparent link between *GSTM1* and *GSTT1* deletion polymorphisms and systolic or diastolic BP control. The patient’s lifestyle seems to be more determining in BP control under treatment than studied genetic factors. Especially alcohol consumption and physical inactivity are associated with a poor control or uncontrolled BP. In addition, given the fact that advanced disease stage, with or without cardiac complications, is also linked to an uncontrolled BP, early diagnosis should be therefore encouraged for effective management and for better therapeutic responses.

## Limitations

Our study could have certain limitations, in particular the size of the study population and the lack of information on adherence to antihypertensive therapy. These observations could be taken into account in future studies.

## Supplementary Information

**Additional file 1: Figure S1.** *GSTM1*, *GSTT1* and *β-globin* genes and corresponding bands in electrophoresis gel. This file shows corresponding bands of *GSTM1*, *GSTT1* and *β-globin* genes in electrophoresis gel. The number 1 through 14 represents individual sample and M represents Molecular weight marker. The strategy to identify presence or absence of *GSTM1*or *GSTT1*was as followed: to validate a PCR product (corresponding to a sample), we must have a band corresponding to *β-globin* and presence or absence of *GSTM1* or *GSTT1* was indicated respectively by the presence or absence of bands corresponding for each gene.

**Additional file 2: Table S1.** Level of treatment and antihypertensive drugs used by patients. This file shows the proportion of antihypertensive drugs used by study participants.

**Additional file 3: Table S2.** Sex and age stratified analysis of association between *GSTM1* and *GSTT1* variants with essential hypertension control. This file shows a stratified analysis of the association between *GSTM1* and *GSTT1* gene variants with essential hypertension control in order to highlight the probable interactions.

**Additional file 4: Table S3.** Age and sex-stratified analysis of non-genetic factors affecting blood pressure control in patients with essential hypertensive. This file shows a stratified analysis by sex and age of non-genetic factors affecting blood pressure control in patients with essential hypertensive to highlight the probable interactions.

**Additional file 5:** Data collection sheet. Information sheet and questionnaire. This file shows the fact sheets used to explain the study during the recruitment of the participants and the questionnaire which served for the data collection.

## Data Availability

The dataset generated in this study is available from NCBI Nucleotide under the accession number LC517160.1.

## Abbreviations

BMI: Body mass index
CERBA: Pietro Annigoni Biomolecular Research Center
DBP: Diastolic blood pressure
EDTA: Ethylenediaminetetraacetic
*GSTM1*: S-transferases Mu 1
*GSTT1*: Glutathione S-transferases Theta 1
HDL-c: High-density lipoprotein cholesterol
LABIOGENE: Laboratory of molecular biology and genetics
LDL-c: Low-density lipoprotein cholesterol
MD: Means difference
PCR: Polymerase chain reaction
ROS: Reactive Oxygen Species
SBP: Systolic blood pressure
SD: Standard deviation
SPSS: Statistical Package for the Social Sciences
TC: Total cholesterol
WC: Waist circumference
