# Supplementary material for: Glutathione S-transferase M1 and T1 genes deletion polymorphisms and blood pressure control among treated essential hypertensive patients in Burkina Faso
Source: BMC Res Notes. 2021 Jun 30;14:244. doi: 10.1186/s13104-021-05658-w (PMC8243756; doi:10.1186/s13104-021-05658-w)
Supplement: Supplementary file 1 — Additional file 1: Figure S1. GSTM1, GSTT1 and β-globin genes and corresponding bands in electrophoresis gel. This file shows corresponding bands of GSTM1, GSTT1 and β-globin genes in electrophoresis gel. The number 1 through 14 represents individual sample and M represents Molecular weight marker. The strategy to identify presence or absence of GSTM1or GSTT1was as followed: to validate a PCR product (corresponding to a sample), we must have a band corresponding to β-globin and presence or absence of GSTM1 or GSTT1 was indicated respectively by the presence or absence of bands corresponding for each gene. [file 13104_2021_5658_MOESM1_ESM.docx]

480 bp (*GSTT1*)

268 bp (*β-globin*)

219 bp (*GSTM1*)

***M****= Molecular weight marker (100bp); GSTM1-active/GSTT1-active=* ***6, 9, 11****; GSTM1-active/GSTT1-null=* ***1, 2, 3, 4, 12, 13****;* *GSTM1-null/GSTT1-active=* ***14****; GSTM1-null/GSTT1 null=* ***5, 7, 8, 10.***

**Additional file 1: Figure S1.** *GSTM1*, *GSTT1*, *β-globin* genes and corresponding bands in electrophoresis gel.
