# Supplementary material for: Glutathione S-transferase M1 and T1 genes deletion polymorphisms and blood pressure control among treated essential hypertensive patients in Burkina Faso
Source: BMC Res Notes. 2021 Jun 30;14:244. doi: 10.1186/s13104-021-05658-w (PMC8243756; doi:10.1186/s13104-021-05658-w)
Supplement: Supplementary file 2 — Additional file 2: Table S1. Level of treatment and antihypertensive drugs used by patients. This file shows the proportion of antihypertensive drugs used by study participants. [file 13104_2021_5658_MOESM2_ESM.docx]

**Additional file 2: Table S1.** level of treatment and antihypertensive drugs used by patients

| Variables | Nombre (200) | Pourcentage (%) |
| --- | --- | --- |
| *Monotherapy* | **99** | **49.5** |
| Amlodipine | 57 | 28.5 |
| Nifedipine | 30 | 15 |
| Atenolol | 3 | 1.5 |
| Captopril | 3 | 1.5 |
| Ramipril | 2 | 1 |
| Enalapril | 4 | 2 |
| *Bitherapy* | **65** | **32.5** |
| IC + ACEI | 10 | 5 |
| IC + D | 7 | 3.5 |
| IC + β-bloquant | 14 | 7 |
| IC + ARA2 | 1 | 0.5 |
| IC + IC | 1 | 0.5 |
| *D* + ACEI | 25 | 12.5 |
| D + ARA2 | 7 | 3.5 |
| *Tritherapy* | **36** | **18** |
| IC + ACEI + D | 27 | 13.5 |
| IC + D + ARA2 | 5 | 2.5 |
| IC + ACEI + AC | 1 | 0.5 |
| ACEI + D + β-bloquant | 1 | 0.5 |
| ACEI + IC + β-bloquant | 2 | 1 |

*Values are expressed in numbers and percentages;* ***IC****: calcium channel blockers;* ***ACEI****: angiotensin converting enzyme inhibitors;* ***D****: diuretics;* ***AC****: central antihypertensive;* ***ARA2****: angiotensin 2 receptor antagonists.*
