# Supplementary material for: Glutathione S-transferase M1 and T1 genes deletion polymorphisms and blood pressure control among treated essential hypertensive patients in Burkina Faso
Source: BMC Res Notes. 2021 Jun 30;14:244. doi: 10.1186/s13104-021-05658-w (PMC8243756; doi:10.1186/s13104-021-05658-w)
Supplement: Supplementary file 3 — Additional file 3: Table S2. Sex and age stratified analysis of association between GSTM1 and GSTT1 variants with essential hypertension control. This file shows a stratified analysis of the association between GSTM1 and GSTT1 gene variants with essential hypertension control in order to highlight the probable interactions. [file 13104_2021_5658_MOESM3_ESM.docx]

| Parameters | Genes -Variants | Systolic Blood Pressure (SBP) | | |  | Diastolic Blood Pressure (DBP) | | |  | Hypertension | | |  |  |
| --- | --- | --- | --- | --- | --- | --- | --- | --- | --- | --- | --- | --- | --- | --- |
|  |  | **Controlled**  ***N* = 126 (%)** | **Uncontrolled**  ***n* = 74 (%)** | ***p* value** |  | **controlled**  ***n* = 152 (%)** | **uncontrolled**  ***n* = 48 (%)** | ***p* value** |  | **Controlled**  ***N* = 115 (%)** | **Uncontrolled**  ***n* = 85 (%)** | ***p* value** | | |
| Male | ***# GSTM1-active*** | 31 (70.45) | 23 (76.67) |  |  | 39 (75.00) | 15 (68.18) |  |  | 29 (72.50) | 25 (73.53) |  | | |
|  | ***GSTM1-null*** | 13 (29.55) | 7 (23.33) | 0.60 |  | 13 (25.00) | 7 (31.82) | 0.57 |  | 11 (27.50) | 9 (26.47) | 1.00 | | |
|  | ***# GSTT1-active*** | 21 (47.72) | 10 (33.33) |  |  | 22 (42.31) | 9 (40.91) |  |  | 20 (50.00) | 11 (32.35) |  | | |
|  | ***GSTT1-null*** | 23 (52.28) | 20 (66.66) | 0.24 |  | 30 (57.69) | 13 (51.09) | 1.00 |  | 20 (50.00) | 23 (67.65) | 0.15 | | |
|  | ***#GSTM1*(*+*)*/GSTT1*(*+*)** | 9 (20.45) | 7 (23.33) |  |  | 12 (23.08) | 4 (18.18) |  |  | 9 (22.50) | 7 (20.59) |  | | |
|  | ***GSTM1*(*-*)*/GSTT1*(*-*)** | 1 (2.27) | 4 (13.33) | 0.31 |  | 3 (5.77) | 2 (9.09) | 0.59 |  | 0 (0.00) | 5 (14.71) | NA | | |
| Female | ***# GSTM1-active*** | 54 (65.85) | 33 (75.00) |  |  | 70 (70.00) | 17 (65.38) |  |  | 50 (66.67) | 37 (72.55) |  | | |
|  | ***GSTM1-null*** | 28 (34.15) | 11 (25.00) | 0.31 |  | 30 (30.00) | 9 (34.62) | 0.64 |  | 25 (33.33) | 14 (27.45) | 0.55 | | |
|  | ***# GSTT1-active*** | 28 (34.15) | 17 (38.64) |  |  | 35 (35.00) | 10 (38.46) |  |  | 25 (33.33) | 20 (39.22) |  | | |
|  | ***GSTT1-null*** | 54 (65.85) | 27 (61.36) | 0.69 |  | 65 (65.00) | 16 (61.54) | 0.81 |  | 50 (66.67) | 31 (60.78) | 0.57 | | |
|  | ***#GSTM1*(*+*)*/GSTT1*(*+*)** | 16 (19.51) | 9 (20.45) |  |  | 22 (22.00) | 3 (11.54) |  |  | 15 (20.00) | 10 (19.61) |  | | |
|  | ***GSTM1*(*-*)*/GSTT1*(*-*)** | 16 (19.51) | 3 (6.82) | 0.18 |  | 17 (17.00) | 2 (7.69) | 1.00 |  | 15 (20.00) | 4 (7.84) | 0.21 | | |
| ≤ 55 years | ***# GSTM1-active*** | 54 (67.50) | 27 (72.97) |  |  | 60 (69.77) | 21 (67.74) |  |  | 48 (68.57) | 33 (70.21) |  | | |
|  | ***GSTM1-null*** | 26 (32.50) | 10 (27.02) | 0.66 |  | 26 (30.23) | 10 (32.26) | 0.82 |  | 22 (31.43) | 14 (29.79) | 1.00 | | |
|  | ***# GSTT1-active*** | 34 (42.50) | 17 (45.95) |  |  | 38 (44.19) | 13 (41.93) |  |  | 30 (42.86) | 21 (44.68) |  | | |
|  | ***GSTT1-null*** | 46 (57.50) | 20 (54.05) | 0.84 |  | 48 (55.81) | 18 (58.07) | 1.00 |  | 40 (57.14) | 26 (55.32) | 0.85 | | |
|  | ***#GSTM1*(*+*)*/GSTT1*(*+*)** | 15 (18.75) | 9 (24.32) |  |  | 23 (26.74) | 4 (12.90) |  |  | 17 (24.28) | 10 (21.28) |  | | |
|  | ***GSTM1*(*-*)*/GSTT1*(*-*)** | 18 (22.50) | 3 (8.10) | 0.10 |  | 11 (12.79) | 1 (2.23) | 1.00 |  | 9 (12.85) | 3 (6.38) | 0.71 | | |
| ˃ 55 years | ***# GSTM1-active*** | 31 (67.39) | 28 (75.68) |  |  | 48 (72.73) | 11 (64.71) |  |  | 31 (68.89) | 28 (75.68) |  | | |
|  | ***GSTM1-null*** | 15 (32.61) | 9 (24.42) | 0.47 |  | 18 (27.27) | 6 (35.29) | 0.55 |  | 14 (31.11) | 9 (24.32) | 0.62 | | |
|  | ***# GSTT1-active*** | 15 (32.61) | 11 (29.73) |  |  | 20 (30.30) | 6 (35.29) |  |  | 15 (33.33) | 10 (27.03) |  | | |
|  | ***GSTT1-null*** | 31 (67.39) | 26 (70.27) | 0.81 |  | 46 (69.70) | 11 (64.71) | 0.77 |  | 30 (66.67) | 27 (72.97) | 0.63 | | |
|  | ***#GSTM1*(*+*)*/GSTT1*(*+*)** | 7 (15.22) | 7 (18.92) |  |  | 11 (16.67) | 3 (17.65) |  |  | 7 (15.56) | 7 (18.92) |  | | |
|  | ***GSTM1*(*-*)*/GSTT1*(*-*)** | 7 (15.22) | 5 (13.51) | 0.71 |  | 9 (13.64) | 3 (17.65) | 1.00 |  | 6 (13.33) | 6 (16.22) | 1.00 | | |

**Additional file 3: Table S2.** Sex and age stratified analysis of association between *GSTM1* and *GSTT1* variants with essential hypertension control
