# Supplementary material for: Glutathione S-transferase M1 and T1 genes deletion polymorphisms and blood pressure control among treated essential hypertensive patients in Burkina Faso
Source: BMC Res Notes. 2021 Jun 30;14:244. doi: 10.1186/s13104-021-05658-w (PMC8243756; doi:10.1186/s13104-021-05658-w)
Supplement: Supplementary file 4 — Additional file 4: Table S3. Age and sex-stratified analysis of non-genetic factors affecting blood pressure control in patients with essential hypertensive. This file shows a stratified analysis by sex and age of non-genetic factors affecting blood pressure control in patients with essential hypertensive to highlight the probable interactions. [file 13104_2021_5658_MOESM4_ESM.docx]

| Parameters |  | Sex | |  | Age group | |
| --- | --- | --- | --- | --- | --- | --- |
|  |  | **Women** | **Men** |  | **≤ 55 years** | **˃ 55 years** |
| Residence |  |  |  |  |  |  |
| Rural/ Urban |  | 1.00 | 0.47 |  | 0.81 | 0.14 |
| Behavioral factors |  |  |  |  |  |  |
| Current alcohol use |  | 0.47 | **0.01*** |  | 0.13 | **0.04*** |
| Current tobacco use |  | 1.00 | 0.40 |  | 0.47 | 1.00 |
| Low sodium diet |  | 0.65 | 0.40 |  | 0.47 | 1.00 |
| Lack of physical exercise |  | 0.80 | 0.46 |  | 0.56 | 1.00 |
| Normal weight |  | 1.00 | 0.65 |  | 0.36 | 0.36 |
| Overweight and obesity |  | 1.00 | 0.65 |  | 0.36 | 0.36 |
| Central obesity |  | 1.00 | 0.83 |  | 0.70 | 0.19 |
| Grade hypertension |  |  |  |  |  |  |
| Grade I |  |  |  |  |  |  |
| Grade II |  |  |  |  |  |  |
| Grade III |  |  |  |  |  |  |
| Personal history |  |  |  |  |  |  |
| Heart involvement Yes/No |  | **0.04*** | 0.10 |  | 0.11 | **0.04*** |
| Diabetes mellitus Yes/No |  | 0.59 | **0.03*** |  | 0.23 | 0.23 |
| Asthma Yes/No |  | 0.46 | 1.00 |  | 1.00 | 1.00 |
| Taste Yes/No |  | 1.00 | 0.64 |  | 0.64 | 1.00 |
| Family history |  |  |  |  |  |  |
| Hypertension Yes/No |  | 0.63 | 1.00 |  | 0.17 | 0.12 |
| Diabetes mellitus Yes/No |  | 0.75 | 0.15 |  | 0.80 | 0.41 |
| Treatment level |  |  |  |  |  |  |
| Monotherapy |  | 0.24 | 0.85 |  | 0.25 | 1.00 |
| Bitherapy |  | 0.06 | 0.83 |  | 0.14 | 1.00 |
| Tritherapy |  | 0.53 | 0.64 |  | 0.77 | 0.80 |
| Professional status |  |  |  |  |  |  |
| Household |  | 0.41 | 0.20 |  | 0.13 | 0.38 |
| Farmer |  | 0.36 | 1.00 |  | 0.21 | 0.24 |
| Official |  | 0.63 | 0.42 |  | 0.42 | 0.28 |
| Daily |  | 1.00 | 0.46 |  | 1.00 | 1.00 |
| Unemployed |  | 1.00 | 0.68 |  | 1.00 | 1.00 |
| Retirement |  | 0.53 | 0.38 |  | 1.00 | 0.55 |
| trader |  | 0.45 | 0.77 |  | 0.62 | 1.00 |
| Other |  | 1.00 | 0.15 |  | 0.64 | 1.00 |

**Additional file 4: Table S3.** Age and sex-stratified analysis of non-genetic factors affecting blood pressure control in patients with essential hypertensive

*Values represent p values of the comparison between controlled and uncontrolled groups using the chi-square test; *: significant difference between the groups (p < 0.05).*
