# Supplementary material for: Glutathione S-transferase M1 and T1 genes deletion polymorphisms and blood pressure control among treated essential hypertensive patients in Burkina Faso
Source: BMC Res Notes. 2021 Jun 30;14:244. doi: 10.1186/s13104-021-05658-w (PMC8243756; doi:10.1186/s13104-021-05658-w)
Supplement: Supplementary file 5 — Additional file 5: Data collection sheet. Information sheet and questionnaire. This file shows the fact sheets used to explain the study during the recruitment of the participants and the questionnaire which served for the data collection. [file 13104_2021_5658_MOESM5_ESM.docx]

**Additional file 5: Data collection sheet**

INFORMATION ON THE STUDY

1. Information note for participants

I am ......................................................... and I am working on a research project whose title is “Management of Essential Arterial Hypertension: Influence of polymorphisms of *ABCB1* and *CYP3A5* genes on the associated blood pressure parameters and cardiovascular risk factors, hypotensive response of patients to the main antihypertensive drugs used in Ouagadougou”.

2. Promoters: CERBA, LABIOGENE, University Joseph Ki-Zerbo

3. Investigators: Herman Karim SOMBIE, Pr Jacques SIMPORE, Dr Hassanata MILOGO, Dr Jonas K. KOLOGO, Dr Florencia DJIGMA

4. Purpose of the study: Characterize the variants of the *ABCB1* and *CYP3A5* genes in hypertensive and normotensive subjects in Burkina Faso.

5. Participant's Freedom: Participation in the study is voluntary and may be canceled at any time, without cause and without incurring any consequences for the participant.

6. Benefits: Your participation in this study is crucial to achieving the objectives set. The smooth running of the study will provide scientists in Burkina Faso and around the world with information on the prevalence of polymorphisms of the *ABCB1* and *CYP3A5* genes and their influence on antihypertensive treatments.

7. Motivation: No money will be given in exchange for your participation. The results of your various exams will be given to you by a doctor or a psychologist without charge.

8. Participant Commitment: The study will consist of a questionnaire followed by a blood sample of approximately 10 ml. Strict adherence to treatment is required.

9. Your personal expenses in this study: You will not have any expenses to do personally during this study.

10. Commitment of the Principal Investigator: As the lead investigator, I undertake to conduct this research work according to ethical and ethical standards, to protect the physical, psychological and social integrity of individuals throughout during this research and to ensure the confidentiality of the information collected. I also pledge to provide participants with all the support necessary to mitigate the negative effects that may result from participation in this study.

11. Management of Biological samples: Samples collected during this study will be stored at -80 ° C for potential studies on essential hypertension. But any subsequent use will only be done after approval of the ethics committee for health research.

12. Confidentiality of information: All information concerning participants will be kept anonymously and confidentially. The data processing is not nominative, it does not enter therefore in the computer law and freedom (the right of access and rectification is not admissible). The transmission of information concerning the participant for the expertise or the scientific publication will also be anonymous.

13. Ethical consideration: The principal investigator undertakes to maintain absolutely confidentiality and professional secrecy for all information concerning the participant.

14. If you have any question, you can ask them now or later. If you wish to do so later, you can contact: Pr Jacques SIMPORE, Director of the Pietro Annigoni Biomolecular Research Center (CERBA) and of the Laboratory of Molecular Biology and Genetics (LABIOGENE)

Dr Jonas K. KOLOGO, Cardiologist, 70 26 83 48

Mr Herman K. SOMBIE, PhD candidate, 76 10 35 74

**FICHE D’ENQUETE POUR LES PATIENTS HYPERTENDUS (3 pages)**

**FICHE DE CONSENTEMENT ECLAIREE**

***CONSENT OF THE PARTICIPANT***

I, the undersigned ............................................................... .., agree to take part in the research project entitled: Management of Essential Arterial Hypertension: Influence of polymorphisms of *ABCB1* and *CYP3A5* genes on the associated blood pressure parameters and cardiovascular risk factors, hypotensive response of patients to the main antihypertensives used in Ouagadougou.

I declare that:

- I have read the information contained in the general information form of the project;

- I was able to ask questions and all my questions had an adequate answer;

- I understand that taking part in the study is voluntary and I have not been forced to do so;

- I also understand that samples collected during the study may be used later in other studies, once the approval of the ethics committee obtained.

Ouagadougou,...........................................................................................................................

Signature of the participant

**…………………………………………………………………………………………………...**

***DECLARATION OF THE INITIATOR OF THE RESEARCH PROJECT***

I, the undersigned ................................................................................................, declare that:

- I explained the general information of the research project to: ...............................................

....................................................................................................................................................;

- I encouraged him to ask questions and I took the time to answer them;

- I am satisfied that he has fully understood all aspects of the research discussed above.

- I undertake to enforce the terms of this consent form in order to carry out this research under the best conditions, reconciling respect for individual rights and freedoms with the requirements of scientific work.

                     Ouagadougou,..........................................................................................................

                     Signature of the initiator of the research project (or his representative)

**FICHE D’ENQUETE POUR LES TEMOINS NORMO-TENDUS (2 pages)**

***OUESTIONNAIRE FOR HYPERTENSIVES (3 pages)***

Sheet n°: …............................….……………......… Patient code:…...……………...………………… Collection Center:…...………………...………….… Date: ……………………...…….………...…...

Last name and first names: …………………….……………………………………………….………..

Phones :……..……………………………………..…………………….…………….…………….

***I. Socio-anthropometric data***

Gender:……/ Age:……..years/ Father's ethnicity:…….………/ Mother’s ethnicity : ………..………/

Place of residence:………………….. /District:……….......……./Profession: ……….………………. /

Weight:………….….….. Kg/ Eight:……..….….Cm/ Waist circumference:……............………Cm/

Economic level: Low /__/ Middle /__/ High /__/

***II. antecedents***

Personnal: Diabetes /__/  Goutte /__/ Asthma /__/ Dyslipidemia/__/

Others :….……………………………………..…………..……..………………..………………..…....

Familial: HTA: Yes/__/ No/__/; if yes, who is it: ……………………..……...…..…………….…..

Diabetes: Yes /__/ No/__/ ; if yes, who is it: …………..……………..……..……………….……..

Asthma Yes/__/ No/__/ ; if yes, who is it: ….………………………..……………………………..

***III. Style of life***

Alcohol: Yes/__/ Quantity: ……...............…….. No/__/ Stop >3ans/__/ <3ans/__/

Tobacco: Yes/__/ Quantity: ...……………….…No/__/ Stop >3ans/__/ <3ans/__/

Salt: Yes/__/ No/__/ low/__/ Coffee/Tea/Cola: Yes /__/ No/__/ Low/__/

Other eating habits: …………………..….……………………………………...............…………

Sport: Yes /__/ No/__/ ; if yes which one:…………………, frequency/week :…………………...

Do you walk regularly?: No/__/ Yes/__/ Frequency or Kilometers/week:…….…………….….….

Moving: Motor/__/ bicycle/__/ car/__/ Foot/__/ sedentary/__/

***IV. Clinical data***

| Dates | SBP | DBP | MAPA |  | Dates | SBP | DBP | MAPA |
| --- | --- | --- | --- | --- | --- | --- | --- | --- |
|  |  |  |  |  |  |  |  |  |
|  |  |  |  |  |  |  |  |  |
|  |  |  |  |  |  |  |  |  |
|  |  |  |  |  |  |  |  |  |

*Electrocardiogram*

ECG normal /__/ Sinus Bradycardia /__/ Sinus tachycardia /__/

Left atrial hypertrophy /__/ Right AH /__/ LVH/__/ RVH/__/ ACFA/__/ Flutter /__/

Left axial deviation /__/ Right axial deviation /__/ Normal heart axis /__/

Left branch block: Completed /__/ uncompleted /__/

Right branch block: Completed /__/ uncompleted /__/

T negative waves /__/ territory: ………….……………………………….…….…………………..

*Cardiac Echo-Doppler*:  Normal /__/

Left Cavities: dilated: Yes/__/ no/__/ DTD VG: …… mm DTS VG:……mm surf OG: ……………………………..………….. DTD OG: …..……...…………………………… mm

SIV/PP: ……………….………………………………………..…………………….……………….….

FEVG Teicholz: …………….……………………………...…………………………………………….

MVGi: ……………………………..…….... MVG surf corp: …….….……………………………..

*Doppler*

IT/__/ IM/__/ IP/__/ PAPS: …..………...…….……………………………mmHg

Pericardial effusion: Present/__/ Absent /__/

| DTDVG : | DTSVG : | SIVd : | Ppd: | FR : | FE : | Aorte : | OG : |
| --- | --- | --- | --- | --- | --- | --- | --- |

*Ocular involvement*: ………………………………………………………………………………..…..

……………..…………………………………………………………………………….………………

***V. Biological data***

| Dates | Exams | Interpretations |
| --- | --- | --- |
|  | -Glucose: -GOT: -GPT:  -Tot-Chol : -HDL : -LDL :  -Triglycerides: -Creatinine : -K^+^:  -Na^+^ : -Mg^2+^: -Cl^-^ :  -Calcium : -Uricemia :  -Proteinuria/24H : |  |

***VI. Treatments***

*Antihypertensive molecules:*

**1^rst^ molecule**: …………………………………………Dose:……..………………………………

Initiation date: ………………………………………………………………………………………..

**2^nd^ molecule** :…………………………………………Dose:……..………………………………

Initiation date:………………………………………………………………………………………..

**3^rd^ molecule** : …………………………………………Dose:……..………………………………

Initiation date: ………………………………………………………………………………………..

**4^th^ molécule** : …………………………………………Dose:……..………………………………

Initiation date: ………………………………………………………………………………………..

*Others molecules*: ……………..………………………………………..……………………………….

………………………………………………………………………….……..…….………………………………………………………………………………………………………………………………………………………………………………………………………………………………………………

………………………………………………….………………………………………………………...

…………………………………….…………………………………………………………………...…

………………………….………………….……………………………………………………....……..

Conclusion: ………………………….……………..………………………………...………………….. ……………………………….……………………………………………………………….…………..

……………………………….………………………………………………………………….………..

……………………………….…………………………………………………………………….……..

………………………………………………………………………………………………….…...……

…………………………………………………………………..……………………………..………………………………………………………………………………………………………………………………………………………………………………………………………………………………….…………………………………………………………………………………………………………………………………………………………………………………………………………………………………………………………………………………………………………………………………………………………………...………………………………………………………….……………………

**FICHE D’ENQUETE POUR LES TEMOINS NORMO-TENDUS (2 pages)**

**OUESTIONNAIRE FOR NORMOTENSIVES**

Sheet n°: …............................….……………......… Patient code:…...……………...………………… Collection Center:…...………………...………….… Date: ……………………...…….………...…...

Last name and first names: …………………….……………………………………………….………..

Phones :……..……………………………………..…………………….…………….…………….

***I. Socio-anthropometric data***

Gender:……/ Age:……..years/ Father's ethnicity:…….………/ Mother’s ethnicity : ………..………/

Place of residence:………………….. /District:……….......……./Profession: ……….………………. /

Weight:………….….….. Kg/ Eight:……..….….Cm/ Waist circumference:……............………Cm/

Economic level: Low /__/ Middle /__/ High /__/

***II. antecedents***

Personnal: Diabetes /__/  Goutte /__/ Asthma /__/ Dyslipidemia/__/

Others :….……………………………………..…………..……..………………..………………..…....

Familial: HTA: Yes/__/ No/__/; if yes, who is it: ……………………..……...…..…………….…..

Diabetes: Yes /__/ No/__/ ; if yes, who is it: …………..……………..……..……………….……..

Asthma Yes/__/ No/__/ ; if yes, who is it: ….………………………..……………………………..

***III. Style of life***

Alcohol: Yes/__/ Quantity: ……...............…….. No/__/ Stop >3ans/__/ <3ans/__/

Tobacco: Yes/__/ Quantity: ...……………….…No/__/ Stop >3ans/__/ <3ans/__/

Salt: Yes/__/ No/__/ low/__/ Coffee/Tea/Cola: Yes /__/ No/__/ Low/__/

Other eating habits: …………………..….……………………………………...............…………

Sport: Yes /__/ No/__/ ; if yes which one:…………………, frequency/week :…………………...

Do you walk regularly?: No/__/ Yes/__/ Frequency or Kilometers/week:…….…………….….….

Moving: Motor/__/ bicycle/__/ car/__/ Foot/__/ sedentary/__/

***IV. Clinical data***

| Dates | SBP | DBP | MAPA |  | Dates | SBP | DBP | MAPA |
| --- | --- | --- | --- | --- | --- | --- | --- | --- |
|  |  |  |  |  |  |  |  |  |
|  |  |  |  |  |  |  |  |  |
|  |  |  |  |  |  |  |  |  |
|  |  |  |  |  |  |  |  |  |

*Electrocardiogramme*

*Electrocardiogram*

ECG normal /__/ Sinus Bradycardia /__/ Sinus tachycardia /__/

Left atrial hypertrophy /__/ Right AH /__/ LVH/__/ RVH/__/ ACFA/__/ Flutter /__/

Left axial deviation /__/ Right axial deviation /__/ Normal heart axis /__/

Left branch block: Completed /__/ uncompleted /__/

Right branch block: Completed /__/ uncompleted /__/

T negative waves /__/ territory: ………….……………………………….…….…………………..

*Cardiac Echo-Doppler*:  Normal /__/

Left Cavities: dilated: Yes/__/ no/__/ DTD VG: …… mm DTS VG:……mm surf OG: ……………………………..………….. DTD OG: …..……...…………………………… mm

SIV/PP: ……………….………………………………………..…………………….……………….….

FEVG Teicholz: …………….……………………………...…………………………………………….

MVGi: ……………………………..…….... MVG surf corp: …….….……………………………..

*Doppler*

IT/__/ IM/__/ IP/__/ PAPS: …..………...…….……………………………mmHg

Pericardial effusion: Present/__/ Absent /__/

| DTDVG : | DTSVG : | SIVd : | Ppd: | FR : | FE : | Aorte : | OG : |
| --- | --- | --- | --- | --- | --- | --- | --- |

*Ocular involvement*: ………………………………………………………………………………..…..

……………..…………………………………………………………………………….………………

***V. Biological data***

| Dates | Exams | Interpretations |
| --- | --- | --- |
|  | -Glucose: -GOT: -GPT:  -Tot-Chol : -HDL-c : -LDL-c :  -Triglycerides: -Creatinine : -K^+^:  -Na^+^ : -Mg^2+^: -Cl^-^ :  -Calcium : -Uricemia :  -Proteinuria/24H : |  |
